# Early age at menarche and history of sexually transmitted infections significantly predict cervical cancer screening uptake among women aged 25–49 years: evidence from the 2021 Côte d’Ivoire demographic and health survey

**DOI:** 10.1186/s12913-024-10881-9

**Published:** 2024-04-03

**Authors:** Joshua Okyere, Castro Ayebeng, Kwamena Sekyi Dickson

**Affiliations:** 1 Department of Population and Health, University of Cape Coast, Cape Coast, Ghana; 2 School of Nursing & Midwifery, College of Health Sciences, Kwame Nkrumah University of Science and Technology, Kumasi, Ghana

**Keywords:** Reproductive health, Cervical cancer, Screening, Sexual health

## Abstract

**Introduction:**

Cervical cancer is the second dominant type of cancer among Ivorian women with an estimated age-standardised incidence and mortality rate of 31.2 cases and 22.8 deaths per 100,000 women in 2020, respectively. The Ivorian government through its Ministry of Health implemented the National Cancer Control Programme (NCCP) in 2003 with the aim of improving the prevention, early detection and treatment of cancers in Côte d’Ivoire. Yet, there is a low uptake of CCS (1.2%). Thus, making CCS uptake an important public health concern in the country. Understanding of the extent to which reproductive factors predict CCS uptake is limited in literature. This study aimed to investigate reproductive factors as a predictor of women’s uptake of CCS in Côte d’Ivoire.

**Methods:**

Data from the 2021 Côte d’Ivoire Demographic and Health Survey. A sample of 9,078 women aged 25–49 years were analyzed. The outcome variable was CCS uptake while other variables considered included age at menarche, history of STI, sexual debut, parity, age, educational level, wealth index, health insurance, place of residence, and media exposure. A multivariable logistic regression model was fitted to examine the association between the outcome of interest and predictors at 95% confidence interval.

**Results:**

Approximately, 7.52% of women aged 25–49 years had ever undergone testing for cervical cancer by a healthcare provider. Early menarche was associated with lower odds of CCS uptake [AOR = 0.78; CI = 0.65–0.95]. Compared to those who had no STI, women with a history of STI were more likely to screen for cervical cancer [AOR = 2.63; CI = 2.02–3.42]. Increasing age, higher educational attainment, having health insurance, and being exposed to media were significantly associated with CCS uptake.

**Conclusion:**

In Cote d’Ivoire, age at menarche and STI history constitute reproductive factors that were significantly associated with women’s uptake of CCS. It is imperative for public policy to focus on increasing CCS in these higher-risk women (i.e., women who experienced early menarche, women with early sexual debut and higher parity) through increased sensitization on cervical cancer risk factors.

**Supplementary Information:**

The online version contains supplementary material available at 10.1186/s12913-024-10881-9.

## Background

Globally, there is ubiquitous consensus that cervical cancer is a serious public health concern [1]. The recognition of cervical cancer as a public health concern was highlighted in the World Health Organisation (WHO) Director’s call to eliminate this disease [2]. Cancer of the cervix is the fourth most common cancer among women worldwide, with an estimated 604,000 new cases and 342,000 deaths reported in 2020 [3]. Within the sub-Saharan African (SSA) context, cervical cancer remains the second most reported cancer among women [4].

The situation in Côte d’Ivoire is not different from what has been found in SSA. Available evidence indicates that cervical cancer is the second dominant type of cancer among Ivorian women with an estimated age-standardised incidence and mortality rate of 31.2 cases and 22.8 deaths per 100,000 women in 2020, respectively [5, 6]. To address the high incidence and mortality attributable to cervical cancer, the Ivorian government through its Ministry of Health implemented the National Cancer Control Programme (NCCP) in 2003 with the aim of improving the prevention, early detection and treatment of cancers in Côte d’Ivoire [7]. A core tenet of the NCCP was to improve women’s utilization of cervical cancer screening (CCS) methods including visual inspection with acetic acid and cryotherapy [6, 7]. Consequently, registered health care facilities in both government and private sector, as well as HIV integrated facilities offer CCS services to women aged 25–55 years by adopting a ‘see-and-treat’ approach [7]. Both the NCCP and the American Cancer Society recommend that women initiate CCS at age 25 years [7, 8].

Despite the implementation of the NCCP, the uptake of CCS remains unacceptably low among Ivorian women. A study by Boni et al. [9] revealed that only 1.2% of women in urban areas of Abidjan had undergone screening for cervical cancer. The low uptake of CCS in Côte d’Ivoire have been attributed to unawareness of CCS, negligence, apprehension regarding positive test results, and concerns about additional costs [9]. Additionally, factors such as age, educational level, wealth status, health insurance coverage, and exposure to the media have been found to significantly predict women’s uptake of CCS [6, 9, 10]. Furthermore, CCS in Côte d’Ivoire is not free; it comes at a cost that tends to be a barrier to screening uptake [6]. However, the current body of literature on CCS uptake in Côte d’Ivoire is silent about the role of reproductive factors in predicting CCS uptake among the general women population.

In the context of this study, reproductive factors include age at menarche, sexual debut, parity, and history of sexually transmitted infections (STIs). Extant literature has documented the intricate relationship between reproductive factors and cervical cancer risk. For instance, Pillai et al. [11] reported in their study that Chlamydia infections significantly increase women’s risk of cervical cancer. Another study conducted in China revealed that lower parity was significantly associated with lower odds of developing cervical cancer while concurrent reproductive tract infections exacerbated the risk of cervical cancer [12]. Similarly, in a case-control study conducted among persons living with cervical cancer, it was revealed that early menarche, early sexual debut (i.e., < 18 years), and high parity (i.e., 3–5 births) was associated with a higher risk of cervical cancer [13]. As a public health concern, it is imperative to gain understanding of how high-risk reproductive factors are associated with CCS uptake in Côte D’Ivoire. To the best of our knowledge, there is currently no published research in Côte d’Ivoire that has investigated the extent to which reproductive factors are associated with CCS uptake. This signifies a critical knowledge gap that must be filled. This study aimed to investigate reproductive factors as a predictor of the uptake of CCS among women aged 25–49 years in Côte d’Ivoire.

## Methods

### Study design and data source

We utilized data from the 2021 Côte d’Ivoire Demographic and Health Survey (DHS), which is part of the broader global DHS series. Specifically, the individual recode file (i.e., CIIR81FL) was used. The primary objective of DHS is to collect nationally representative data from developing countries, with a specific focus on women aged 15 to 49 years [14]. To ensure comprehensive national representation, the DHS employed a two-stage sampling design and computed corresponding sampling weights [14].

At the first stage of the sampling, 539 clusters, 261 of which are located in the in urban areas and 278 in rural areas were drawn for survey [15]. A sample of 15,092 households (7,308 urban and 7,784 rural) was chosen, with 28 households selected per cluster [15]. The clusters were selected systematically, with the probability of selection proportional to their household size. These clusters were initially established during the census mapping database conducted in 2019 by the National Institute of Statistics as part of the preparations for the 2021 Population and Housing Census (RGPH) [15]. Data collection was conducted from September 8 to December 30, 2021, by 196 investigating officers organized into 24 teams [15]. All 539 clusters were thoroughly investigated, resulting in the selection of a total of 15,093 households, out of which 14,873 were occupied [15]. Among these occupied households, 14,766 were successfully surveyed, indicating a 99% response rate. Details of the DHS can be found here: https://www.dhsprogram.com/pubs/pdf/FR385/FR385.pdf.

### Study population

**Fig. 1:**
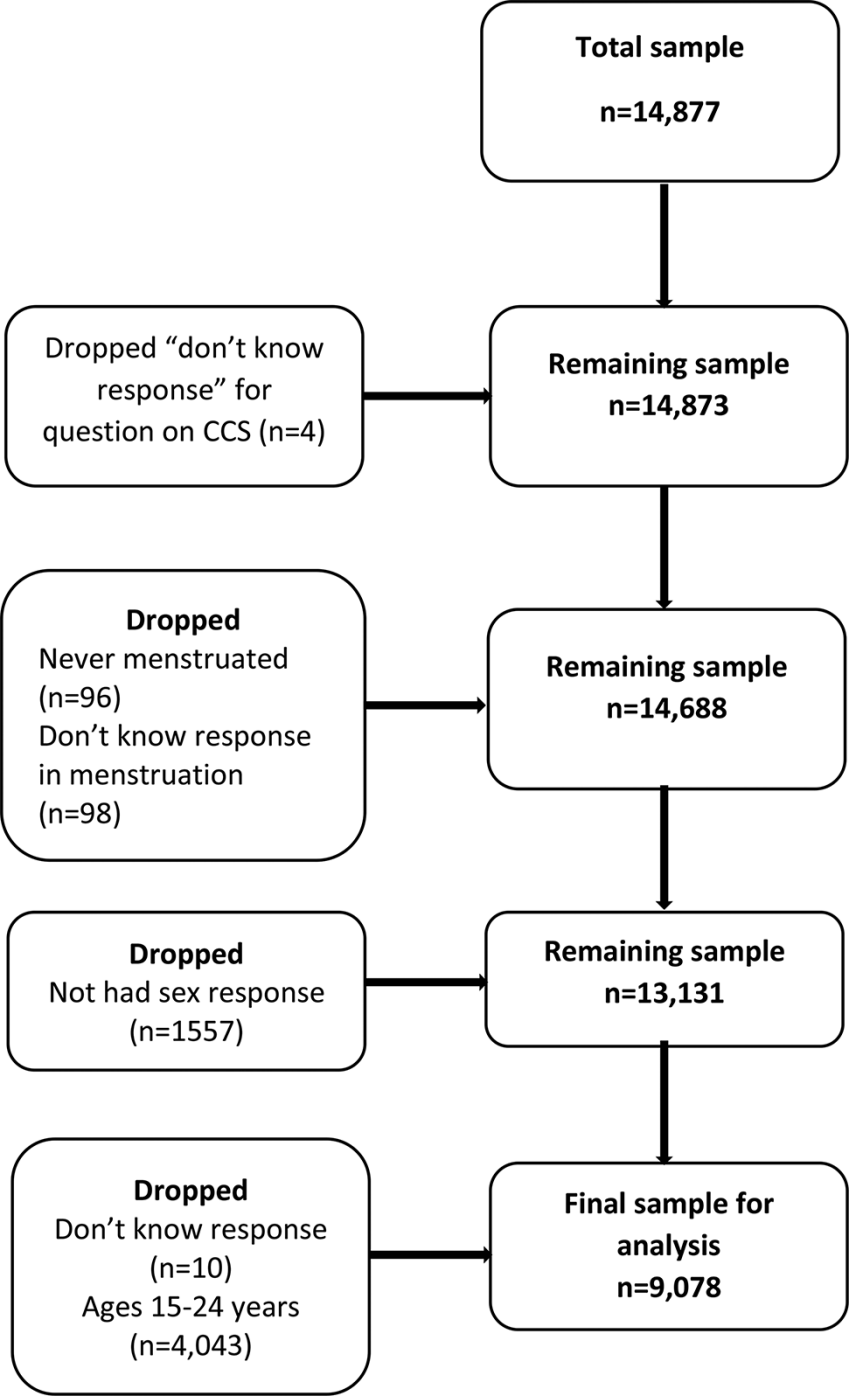
Flowchart of the sampling procedure

As earlier indicated, the 2021 Côte d’Ivoire DHS surveyed a total of 14,766 women aged 15–49 years. However, for this study, we excluded women younger than 25 years as per the CCS recommendations [7, 8]. Also, because we were interested in sexual debut and age at menarche, we excluded all women who had never had sex, those who had never menstruated, or did not know the age which they first menstruated. Consequently, our study population was women aged 25–49 years, who had complete data on all variables of interest in this study (see Fig. 1).

### Measures

#### Outcome variable

The dependent variable was CCS utilization which was generated from the question, “Have you ever been tested for cervical cancer by a healthcare provider?”. The responses to this question were no, yes, and don’t know. However, we dropped “don’t know” to have a binary response of “0 = No” and “1 = Yes”.

#### Key explanatory variables

Our key explanatory variable was reproductive factors. This included age at menarche, sexual debut, parity, and history of STIs in the last 12 months. Age at menarche was coded as “before age 15” and “at 15 years or older”. Sexual debut was coded as “before age 18” and “at 18 years or older”, while parity was coded as nulliparous, uniparous and multiparous. History of STIs in the last 12 months had the responses of “Yes” and “No”.

#### Covariates

Informed by a plethora of extant literature [6, 9, 10], a total of eight variables were selected as covariates. This encompassed age, educational level, wealth index, place of residence, frequency of reading newspapers/magazines, frequency of listening to radio, frequency of watching television, health insurance coverage, and marital status (see Supplementary File 1).

### Statistical analyses

The total sample from the data was 14,877. However, after dropping the sample aged 15–24 years, we had a remaining sample of 9,078 (see Fig. 1). We initiated the analysis by applying the STATA weighting the data using the sample weight (v005). Subsequently, we conducted a cross-tabulation to examine the distribution of all sample variables. To assess whether the proportional distribution significantly differed, we computed Pearson’s chi-square (*X*^2^) test.

Next, we employed a bivariable logistic regression model to explore the association between the respective reproductive factors and CCS uptake. The results from the bivariable analysis were presented in terms of odds ratios at a 95% confidence interval. Following the bivariable analysis, we performed a multivariable logistic regression analysis, aiming to adjust for the influences of covariates. The outcomes of the multivariable analysis were presented in the form of adjusted odds ratios and their associated 95% confidence intervals. All these statistical analyses were conducted using STATA version 14 (StataCorp, College Station, TX, USA). We relied on the Akaike information criterion (AIC) to select the best-fit model. In both models, we computed the AIC. The model with the least AIC was selected as the best-fit model, which in this case was Model II.

## Results

### Distribution of CCS uptake across the various explanatory variables

Table 1 shows the distribution of CCS uptake across the various variables. The results indicate that only 7.52% of women aged 25–49 years had ever undergone testing for cervical cancer by a healthcare provider. Regarding the reproductive factors, the proportion of CCS uptake was significantly high among those who menstruated at age 15 or older (8.05%), those who had an STI in the last 12 months (13.55%), those who had their first sex at age 18 or older (11.02%), and among uniparous women (13.96%). Higher proportion of CCS uptake was reported among women aged 45–49 years (9.56%), those residing in urban areas (10.75%), and women with higher educational attainment (31.20%). Also, a higher uptake of CCS was found among women who read the newspaper at least once a week (21.41%), those who listened to the radio at least once a week (12.83%), and those who watched television at least once a week (10.92%). The uptake of CCS was significantly high among women in the richest wealth index (18.21%) and those with health insurance coverage (27.97%).

**Table 1:** Distribution of CCS uptake across the various variables

Variables	Weighted Sample n (%)	Proportion screened n (%)	Chi-square (X^2^); p-value
Age at first menstruation			**X2 = 9.4258; p = 0.002**
Before age 15	5843 (64.36)	423 (7.23)	
At age 15 and above	3235 (35.64)	260 (8.05)	
**Had STI in the last 12 months**			***X***^**2**^ **= 96.9843;*p* < 0.001**
No	8324 (91.69)	581 (6.98)	
Yes	754 (8.31)	102 (13.55)	
**Sexual debut**			***X***^**2**^ **= 28.8213;*p* < 0.001**
Before 18 years	6623 (72.96)	413 (6.23)	
At age 18 year or older	2455 (27.04)	270 (11.02)	
**Parity**			***X***^**2**^ **= 93.9127; *p* < 0.001**
Nulliparous	637 (7.02)	87 (13.67)	
Uniparous	1129 (12.44)	158 (13.96)	
Multiparous	7312 (80.54)	438 (5.99)	
**Age**			*X*^2^ = 7.9698; *p* < 0.093
25–29 years	2331 (25.68)	164 (7.03)	
30–34 years	2357 (25.96)	183 (7.77)	
35–39 years	1972 (21.72)	130 (6.57)	
40–44 years	1488 (16.39)	117 (7.89)	
45–49 years	931 (10.25)	89 (9.56)	
**Place of residence**			***X***^**2**^ **= 137.5343;*p* < 0.001**
Urban	5167 (56.92)	555 (10.75)	
Rural	3911 (43.08)	128 (3.26)	
**Educational level**			***X***^**2**^ **= 492.2283;*p* < 0.001**
No education	5379 (59.25)	186 (3.47)	
Primary	1837 (20.24)	133 (7.24)	
Secondary	1336 (14.72)	200 (14.93)	
Higher	526 (5.79)	164 (31.20)	
**Frequency of reading newspaper/magazine**			***X***^**2**^ **= 208.8969;*p* < 0.001**
Not at all	8052 (88.70)	478 (5.96)	
Less than once a week	566 (6.24)	105 (18.51)	
At least once a week	459 (5.06)	98 (21.41)	
**Frequency of listening to radio**			***X***^**2**^ **= 87.6613;*p* < 0.001**
Not at all	5602 (61.71)	329 (5.88)	
Less than once a week	1863 (20.53)	147 (7.86)	
At least once a week	1613 (17.77)	207 (12.83)	
**Frequency of watching television**			***X***^**2**^ **= 143.5062;*p* < 0.001**
Not at all	3128 (34.46)	84 (2.67)	
Less than once a week	1259 (13.87)	87 (6.92)	
At least once a week	4691 (51.67)	512 (10.92)	
**Wealth index**			***X***^**2**^ **= 399.7699;*p* < 0.001**
Poorest	1744 (19.21)	32 (1.81)	
Poorer	1684 (18.55)	47 (2.82)	
Middle	1706 (18.79)	91 (5.32)	
Richer	1919 (21.14)	144 (7.53)	
Richest	2026 (22.31)	369 (18.21)	
**Health insurance coverage**			***X***^**2**^ **= 413.7774;*p* = 0.001**
Not covered	8337 (91.84)	476 (5.71)	
Covered	741 (8.16)	207 (27.97)	
**Marital status**			***X***^**2**^ **= 53.1469;*p* < 0.001**
Never married	946 (10.42)	121 (12.84)	
Currently in union	7390 (81.40)	505 (6.83)	
Previously in union	742 (8.18)	57 (7.62)	
**Total**	**9078 (100)**	**683 (7.52)**	

### Association between reproductive factors and CCS uptake among women

In Table 2, we present the results from the bivariable and multivariable logistic regression. In the bivariable regression, all four reproductive factors were significant predictors of women’s uptake of cervical cancer screening (CCS). However, after adjusting for the covariates in Model II, two reproductive factors remained significant predictors. Early menarche was associated with lower odds of CCS uptake [AOR = 0.78; 95%CI = 0.64–0.95]. Conversely, compared to those who had no STIs, women with a history of STI were more likely to be screened for cervical cancer [AOR = 2.62; CI = 2.01–3.40]. Additionally, the covariates showed significant associations with CCS uptake: higher educational attainment [AOR = 3.31; CI = 2.26–4.85], older age [AOR = 2.01; CI = 1.43–2.81], belonging to the richest wealth index [AOR = 2.74; CI = 1.68–4.45], and having health insurance coverage [AOR = 2.60; CI = 1.98–3.41] were associated with higher CCS uptake. Moreover, listening to the radio at least once a week was significantly associated with a higher likelihood of CCS uptake compared to not listening to the radio at all [AOR = 1.48; CI = 1.16–1.89].

**Table 2:** Association between reproductive factors and CCS uptake among women

Variables	Model I Odds Ratio (OR)	Model II Adjusted Odds Ratio (AOR)
**Age at first menstruation**		
Before age 15 years	0.81 [0.67–0.97]*	0.78 [0.64–0.95]*
At 15 years and above	Ref.	Ref.
**Had STI in the last 12 months**		
No	Ref.	Ref.
Yes	2.86 [2.24–3.64]***	2.62 [2.01–3.40]***
**Sexual debut**		
Before 18 years	Ref.	Ref.
At 18 years and above	1.36 [1.11–1.65]**	1.02 [0.86–1.31]
**Parity**		
Nulliparous	Ref.	Ref.
Uniparous	0.93 [0.67–1.31]	1.02 [0.71–1.48]
Multiparous	0.44 [0.33–0.59]***	0.85 [0.58–1.23]
**Covariates**		
**Educational level**		
No education		Ref.
Primary		1.75 [1.36–2.25]***
Secondary		2.28 [1.71–3.04]***
Higher		3.31 [2.26–4.85]***
**Age**		
25–29 years		Ref.
30–34 years		1.19 [0.90–1.58]
35–39 years		1.46 [1.09–1.96]*
40–44 years		1.57 [1.14–2.16]**
45–49 years		2.01 [1.43–2.81]***
**Place of residence**		
Urban		Ref.
Rural		0.86 [0.67–1.11]
**Frequency of reading newspaper/magazine**		
Not at all		Ref.
Less than once a week		1.35 [0.99–1.84]
At least once a week		0.87 [0.59–1.28]
**Frequency of listening to radio**		
Not at all		Ref.
Less than once a week		0.92 [0.71–1.19]
At least once a week		1.48 [1.16–1.89]**
**Frequency of watching television**		
Not at all		Ref.
Less than once a week		1.64 [1.17–2.31]**
At least once a week		1.13 [0.82–1.55]
**Wealth index**		
Poorest		Ref.
Poorer		1.19 [0.79–1.81]
Middle		1.69 [1.12–2.57]*
Richer		2.13 [1.36–3.34]**
Richest		2.74 [1.68–4.45]***
**Health insurance coverage**		
Not covered		Ref.
Covered		2.60 [1.98–3.41]***
**Marital status**		
Never married		Ref.
Currently in union		0.78 [0.57–1.05]
Previously in union		0.84 [0.55–1.27]
***Model Fit statistic***		
**Constant**	0.11 [0.08–0.15]***	0.02 [0.01–0.03]***
**Pseudo R** ^**2**^	0.0384	0.1517
**Prob > chi2**	< 0.001	< 0.001
**AIC**	3976.264	3551.073

* *p* < 0.05, ** *p* < 0.01, *** *p* < 0.001; AIC: Akaike Information Criterion; Ref: reference category

## Discussion

Efficient and systematic screening for precancerous lesions and early detection play a critical role in the prevention and treatment of cervical cancer. This study examined the association between reproductive factors (i.e., age at menarche, history of sexually transmitted infections, early initiation of sexual activity, and parity) and the uptake of CCS among Ivorian women aged 25–49 years. Indeed, the study reveals a significant association between reproductive factors including other covariates (such as education, age, exposure to mass media, wealth status, and health insurance coverage) and CCS uptake. Overall, we found that only 7.52% of the sampled population had ever been screened for cervical cancer. Similarly, low CCS uptake has been reported in other studies conducted in Tanzania [16] and Uganda [17] which found 6% and 4.8%, respectively. The observed prevalence is, however, higher when compared to Boni et al. [9] study that reported a prevalence of 1.2%. A probable reason for this difference is that Boni et al.’ study [9] focused only on urban areas of Abidjan while the present study provides a more nationally representative estimation of CCS uptake.

Age at menarche emerged as significant a predictor of women’s uptake of CCS. Our study indicates that women who experienced early menarche (i.e., before 15 years) were less likely to undergo screening for cervical cancer. This result is inconsistent with Sharma and Pattanshetty [13] whose study suggests that early menarche is a high-risk factor for cervical cancer, and thus, a potential factor in influencing CCS uptake. It is unclear what factors contribute to the observed association. Further research into the specific barriers faced by women with early menarche in accessing CCS could provide valuable insights for targeted interventions aimed at increasing screening uptake in this group.

The study showed a strong positive association between women’s history of sexually transmitted infections (STIs) and the likelihood of screening for cervical cancer. Women who had been diagnosed with any STIs within the last 12 months preceding the survey were 2.62 times more likely to undergo screening. Extant literature suggests that STIs such as chlamydia and human papillomavirus (HPV) increase the risks of cervical carcinogenesis [18–20]. We, therefore, postulate that healthcare providers would be more likely to suggest CCS to women who had tested positive for any STIs [21].

As expected, higher educational attainment was associated with a greater likelihood of CCS uptake. This finding aligns with a study conducted in Zimbabwe [22] where women with secondary and tertiary education were 9.4 and 59.4 times more likely to undergo screening, respectively, than those without any formal education. Women with a higher level of education are likely to possess a more comprehensive understanding of the significance of preventive measures like screening for cervical cancer. Consistent with previous studies conducted in Cameroon [10], Burkina Faso [23] and South Africa [24], we found a pattern of increasing screening uptake as age increases. Available evidence suggests that the risks of non-communicable diseases including cervical cancer increase with ageing [25, 26]. Therefore, older women of reproductive age may perceive themselves as being at a higher risk of cervical cancer than younger women. Hence, informing their screening uptake behavior. It is also possible that as age progresses women would have had more opportunity to participate in CCS in their lifetime compared to younger women.

Congruent with existing literature [27–29], the study demonstrated that being exposed to the mass media such as newspapers/magazines, and the radio at least once a week had a positive influence on women’s screening behavior. The result epitomizes the role of the media as a channel for the dissemination of health education messages, encompassing the advantages of participating in adopting preventive health behaviors, including CCS uptake.

We also observed an increased odds of CCS uptake among women of higher wealth status compared to those in the poorest wealth status. This finding aligns with the outcomes of similar research conducted in Cameroon [10] and Kenya [29]. It suggests that economic factors significantly influence women’s access to preventive healthcare services like CCS. Women with greater financial resources may have more opportunities to access and afford healthcare, including screenings. This assertion is further corroborated by our findings that women who had health insurance coverage were more likely to get screened for cervical cancer than those who did not have health insurance. Similar findings have been reported in a South African study [30] that found health insurance coverage to be associated with a 60.3% higher uptake of CCS. Accessing screening for cervical cancer comes with both direct and indirect costs (e.g., transportation costs). However, health insurance coverage offsets the direct cost and reduces the financial barriers to screening uptake.

### Implications for policy and practice

Based on the findings from the study, it is imperative for the Ministry of Health and all healthcare facilities providing CCS services to prioritize the reproductive factors of women as a key indicator for screening. With the exception of a history of STI, women at higher risk for cervical cancer (i.e., women with higher parity, early sexual debut, and early menarche) are not more likely to be screened when accounting for socio-demographic and economic variables. Priority should be given to targeting women who exhibit factors associated with higher risk of cervical cancer but are less likely to undergo screening, such as higher parity, early sexual debut, and early menarche. Our study results substantiate the notion that the implementation of a universal health insurance scheme aimed at ensuring equitable access to healthcare can significantly augment the likelihood of women’s utilization of CCS. The result from this study also highlights how important the media can be leveraged to disseminate information and encourage Ivorian women to undergo screening for cervical cancer. One study [31] has shown that the adoption of entertainment-education approaches such as the use of soap-operas are effective tools for raising women’s awareness about cervical cancer, risk factors, and the need for screening. Similar approaches can be adopted in Cote d’Ivoire to facilitate the leverage of the media to enhance CCS uptake. Countries like Ghana have what is known as the School Health Education Programme (SHEP) where health practitioners are assigned to schools as SHEP coordinators [32]. They are responsible for health promotion and health education at various levels. Cote d’Ivoire’s Ministry of Health can adopt such initiative to ensure that in-school women are reached with CCS information while working to on the side to reach those currently not in school.

### Strengths and limitations of the study

This study boasts several strengths that enhance its validity. We employed a secondary analysis of the recent Demographic and Health survey data, which provides a representative sample of Ivorian women, enhancing the generalizability of our findings. The comprehensive analysis of various reproductive factors by adjusting for other relevant socio-demographic and economic determinants in relation to CCS contributes to a more holistic understanding of this critical public health issue. Additionally, the alignment of our results with existing literature from different regions and countries enhances the credibility and robustness of our findings. However, this study has certain limitations to consider when interpreting the findings. Firstly, the data utilized is cross-sectional, which limits our ability to establish causal relationships between the variables studied. Furthermore, the data relies on self-reported information, which may introduce recall bias or social desirability bias. Also, the study lacks qualitative insights that could provide a deeper understanding of the reasons behind the observed associations, including the influence of some cultural norms and values on screening behavior.

## Conclusion

In Cote d’Ivoire, age at menarche and STI history constitute reproductive factors that were significantly associated with women’s uptake of CCS. It is imperative for public policy to focus on increasing CCS in these higher risk women (i.e., women who experienced early menarche, women with early sexual debut and higher parity) through increased sensitization on cervical cancer risk factors. Also, the Ivorian government and the Ministry of Health should consider expanding their health insurance scheme to cover the cost of CCS. This is likely to significantly narrow the disparities posed by wealth inequalities and non-health insurance coverage.

### Electronic supplementary material

Below is the link to the electronic supplementary material.

Supplementary Material 1

## Data Availability

The datasets generated and/or analysed during the current study are available in the Measure DHS repository: http://dhsprogram.com/data/available-datasets.cfm.

## Abbreviations

AOR: Adjusted Odds Ratio
CI: Confidence Interval
CCS: Cervical Cancer Screening
DHS: Demographic and Health Survey
NCCP: National Cancer Control Programme
