# Supplementary material for: Early age at menarche and history of sexually transmitted infections significantly predict cervical cancer screening uptake among women aged 25–49 years: evidence from the 2021 Côte d’Ivoire demographic and health survey
Source: BMC Health Serv Res. 2024 Apr 3;24:423. doi: 10.1186/s12913-024-10881-9 (PMC10993584; doi:10.1186/s12913-024-10881-9)
Supplement: Supplementary file 1 — Supplementary Material 1 [file 12913_2024_10881_MOESM1_ESM.docx]

**Appendix I: Coding of covariates**

| **Variable Name** | **Code** |
| --- | --- |
| Age | 1=25-29 years |
|  | 2=30-34 years |
|  | 3=35-39 years |
|  | 4=40-44 years |
|  | 5=45-49 years |
| Place of Residence | 0=Urban |
|  | 1=Rural |
| Educational Level | 0=No education |
|  | 1=Primary |
|  | 2=Secondary |
|  | 3=Higher |
| Frequency of Reading Newspaper/Magazine | 0=Not at all |
|  | 1=Less than once a week |
|  | 2=At least once a week |
| Frequency of Listening to the Radio | 0=Not at all |
|  | 1=Less than once a week |
|  | 2=At least once a week |
| Frequency of Watching Television | 0=Not at all |
|  | 1=Less than once a week |
|  | 2=At least once a week |
| Wealth Index | 0=Poorest |
|  | 1=Poorer |
|  | 2=Middle |
|  | 3=Richer |
|  | 4=Richest |
| Health insurance coverage | 0=Not covered |
|  | 1=Covered |
| Marital status | 0=Never married |
|  | 1=Currently in union |
|  | 2=Previously in union |
